# Morphology, Surface Structure and Water Adsorption Properties of TiO_2_ Nanoparticles: A Comparison of Different Commercial Samples

**DOI:** 10.3390/molecules25204605

**Published:** 2020-10-10

**Authors:** Lorenzo Mino, Chiara Negri, Rosangela Santalucia, Giuseppina Cerrato, Giuseppe Spoto, Gianmario Martra

**Affiliations:** 1 Department of Chemistry and NIS Centre, University of Torino, via Giuria 7, 10125 Torino, Italy; chiara.negri@unito.it (C.N.); rosangela.santalucia@unito.it (R.S.); giuseppe.spoto@unito.it (G.S.); gianmario.martra@unito.it (G.M.); 2 Department of Chemistry, Center for materials science and nanotechnology, University of Oslo, Sem Sælands vei 26, 0371 Oslo, Norway

**Keywords:** OH groups, CO adsorption, surface hydration, Lewis acidity, NIR spectroscopy, FT-IR spectroscopy

## Abstract

Water is a molecule always present in the reaction environment in photocatalytic and biomedical applications of TiO_2_ and a better understanding of its interaction with the surface of TiO_2_ nanoparticles is crucial to develop materials with improved performance. In this contribution, we first studied the nature and the surface structure of the exposed facets of three commercial TiO_2_ samples (i.e., TiO_2_ P25, SX001, and PC105) by electron microscopy and IR spectroscopy of adsorbed CO. The morphological information was then correlated with the water adsorption properties, investigated at the molecular level, moving from multilayers of adsorbed H_2_O to the monolayer, combining medium- and near-IR spectroscopies. Finally, we assessed in a quantitative way the surface hydration state at different water equilibrium pressures by microgravimetric measurements.

## 1. Introduction

Titanium dioxide (TiO_2_) is one of the key semiconductor oxides employed to face energetic and environmental issues. In most of its applications TiO_2_, mainly employed in the anatase crystal phase, is immersed in water solutions (e.g., photocatalytic water decontamination, biomedical applications) or is in contact with H_2_O vapor (e.g., photodegradation of air pollutants), and therefore the investigation of H_2_O/TiO_2_ interactions is of paramount importance [1,2]. Several studies with the aim of elucidating at the molecular level the structure of adsorbed water at TiO_2_ surfaces are based on single crystals, mainly focusing on the (101) surface which is the most stable for the anatase polymorph [3]. Among the available surface science techniques, scanning tunneling microscopy (STM) has been frequently employed, owing to its ability to provide direct information about geometry and electronic structure of adsorbed H_2_O [4,5]. Other experimental techniques used to study the H_2_O/TiO_2_ interface in single crystals include temperature-programmed desorption (TPD) [6], surface X-ray diffraction [7], and X-ray photoelectron spectroscopy (XPS) [8]. Often, the interpretation of the experimental results is assisted by density functional theory (DFT) and (ab initio) molecular dynamics calculations [2,9,10].

Even though the above-mentioned surface science studies of single crystal models have provided valuable insights into the nature of H_2_O/TiO_2_ interactions, the extension of these results to “real” high surface area systems employed in the majority of practical applications, is not straightforward. This is due to the complexity of TiO_2_ nanoparticles (NPs), which show a combination of different facets, edges, corners, defects, and often contain other residual species derived from the synthesis (surface or bulk ones) [11]. In this contribution, we applied a surface science approach to three widely employed commercial samples of TiO_2_ NPs (i.e., TiO_2_ P25 by Evonik, SX001 by Solaronix and CristalACTiV™ PC105 by Cristal), trying to correlate their surface structure with their water adsorption properties. Indeed, the presence of a different distribution of surface sites, resulting in a different amount and structure of adsorbed water, can critically influence the photocatalytic activity of the TiO_2_ NPs, as already reported, for instance, for the photodegradation of phenol, acetic acid, and benzoic acid [12,13,14].

In particular, we combined transmission electron microscopy and IR spectroscopy of adsorbed carbon monoxide to determine the main exposed facets in the TiO_2_ NPs [15,16]. Subsequently, we performed an IR spectroscopic study in the medium (MIR) and near (NIR) infrared spectral ranges, monitoring the variations in the water-water and water-TiO_2_ interaction at decreasing coverages from H_2_O multilayers to the monolayer. Indeed, IR spectroscopies have proven to be extremely valuable tools to assess the speciation and surface structure of adsorbed water and hydroxyls at oxide surfaces [17,18,19,20], as well as to monitor the surface processes occurring during photocatalytic reactions [21,22,23]. A quantitative investigation of the amount of H_2_O present at the oxide surface at different water equilibrium pressures was also carried out by microgravimetry.

## 2. Results and Discussion

### 2.1. Particle Morphology and Surface Structure

**Figure 1:**
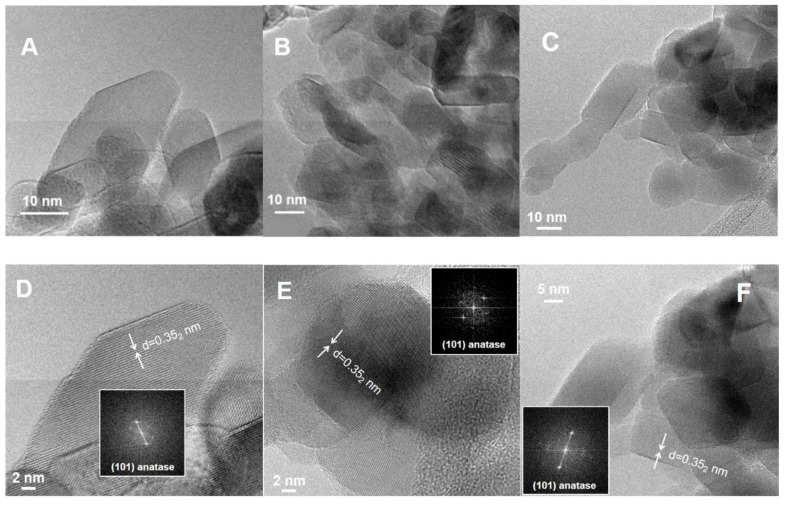
HR-TEM images and related Fourier Transform of selected nanoparticles for TiO_2_ SX001 (panels **A** and **D**), PC105 (panels **B** and **E**) and P25 (panels **C** and **F**).

Before studying the adsorption of water on the activated sample surfaces, we investigated the nanoparticle morphology by HR-TEM and the exposed surface sites by IR spectroscopy of adsorbed CO. Figure 1 reports the micrographs of the three selected samples. In all materials we can find nanoparticles showing a truncated bipyramidal shape, as predicted by the Wulff construction for anatase TiO_2_. However, the truncated bipyramids are not regular and well defined as observed in shape engineered TiO_2_ samples [24,25]. Indeed, the size and shape distribution is heterogeneous and the borders of the NPs are quite irregular. Moreover, the PC105 sample (panel B in Figure 1) shows the presence of aggregates constituted by small nanocrystals. From the Fourier transform analysis of selected nanoparticles (Figure 1D–F), we can find several exposed {101} terminations, however the irregularity of the sample makes it difficult to draw statistically significant conclusions employing only electron microscopy. 

The NPs surface has been further investigated employing CO as probe molecule to get deeper insights on the mainly exposed terminations. Indeed, when adsorbed on TiO_2_, the CO stretching frequency is directly linked to the electrophilicity, and thus to the Lewis acidity, of the surface Ti^4+^ centres located on extended surfaces and defect sites. Moreover, carbon monoxide is not only a very useful probe of the individual Ti^4+^ sites, but can also help to identify the exposed crystal facets when the interpretation of the IR spectra is supported by DFT calculations [3,26] and by experimental data obtained on single crystals [27,28].

**Figure 2:**
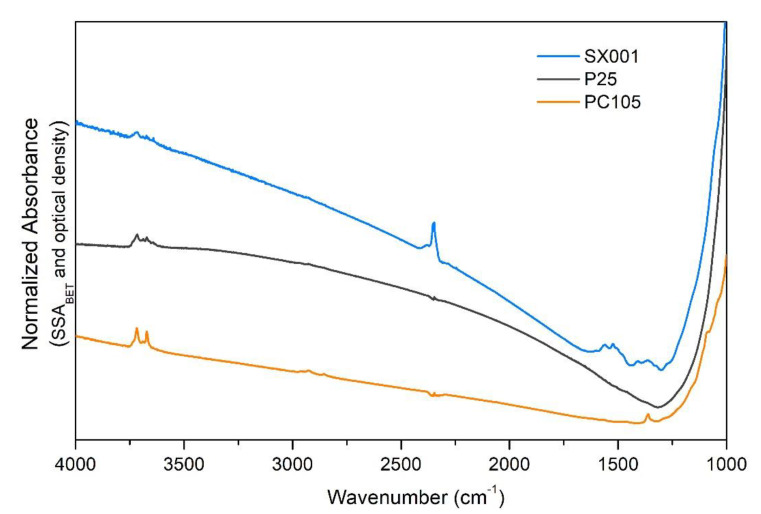
MIR spectra of SX001 (light blue), P25 (dark grey), PC105 (orange) after activation at 873 K. All the spectra have been normalized by dividing the absorbance by the specific surface area (m^2^/g) and the “optical thickness” (g/m^2^) of the pelletized samples.

**Figure 3:**
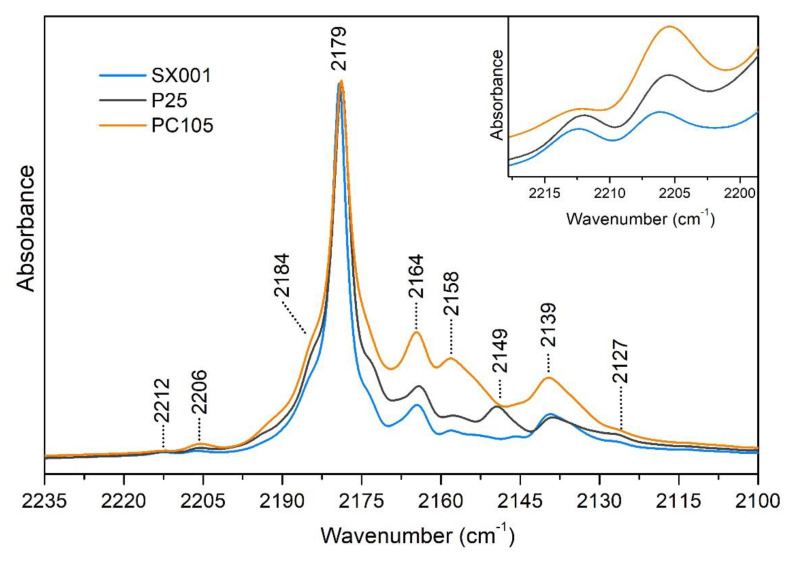
MIR spectra, recorded at 100 K, of CO adsorbed at 25 mbar on SX001 (light blue), P25 (dark grey) and PC105 (orange), previously activated at 873 K. All the spectra have been normalized to the most intense peak at 2179 cm^−1^. In the inset a magnification of the signals showing the higher hypsochromic shift in the CO stretching vibration is reported.

Before studying CO adsorption, the TiO_2_ samples were outgassed at high temperature (see Material and Methods section) to remove water and other adsorbed molecules from the NPs surface. Figure 2 shows the surface picture of the activated samples provided by the MIR spectra collected immediately after the thermal treatment at 873 K. Besides the bands around 3700 cm^−1^, associated with few residual surface OH groups, we can notice that, in the case of PC105, some residual adsorbed sulphates species are present, as evidenced by the peak at 1360 cm^−1^. The presence of sulphates could affect the interaction with molecularly adsorbed water owing to the increase of Lewis acidity of Ti^4+^ centers. However, as we will discuss in the following, the IR spectrum of CO adsorbed on PC105 (orange curve in Figure 3) does not show remarkable differences with respect to the other samples which do not expose sulphate groups, therefore the sulphates surface concentration should be negligible.

Moreover, in the case of SX001 we can observe a complex pattern in the 1765–1215 cm^−1^ region, due to residual carboxylates/carbonates groups, resulting from the calcination of organic moieties of the Ti precursor used for the synthesis and a peak at 2345 cm^−1^, assigned to linearly adsorbed CO_2_. The fact that both species resisted the initial oxidative and outgassing conditioning of the sample clearly indicates that they are trapped in internal cavities of the material, typically created during the formation of particles via sol-gel synthesis.

Upon CO adsorption, we can observe (Figure 3) that all the IR spectra are dominated by a band at 2179 cm^−1^, ascribed to the stretching vibration of CO molecules adsorbed on {101} surfaces [26,27]. The full width at half maximum (FWHM) of the main peak decreases in the order PC105 > P25 > SX001, suggesting the presence of more regular {101} facets in the SX001 sample. The signals at 2212 (see inset of Figure 3) and 2127 cm^−1^ are also related to CO molecules adsorbed on {101} facets: the former is assigned to the combination of ν(CO) with frustrated translational CO modes on TiO_2_, while the latter is due to the internal stretching mode of ^13^CO molecules, present in natural abundance. We can note also some peaks at frequency higher than 2180 cm^−1^, which are more evident in the PC105 sample. These signals are assigned to the presence of CO adsorbed on {110} or {112} minority facets at 2184 cm^−1^ and to Ti centers with high coordinative unsaturation (and high Lewis acidity) at 2206 cm^−1^ [29]. The peak at 2164 cm^−1^ is ascribed to {100} facets. At 2158 cm^−1^ we can observe the band arising from CO interacting with the few hydroxyls groups still remaining after activation at 873 K (see Figure 2). Moreover, a small contribution of CO adsorbed on (1 × 4) reconstructed {001} facets [11] which show a very low polarizing power, could also be present. Only in TiO_2_ P25 is there evident a peak at 2149 cm^−1^, due to the fraction of rutile phase (*ca.* 20%) present in the sample [30]. Finally, the signal due to physisorbed CO is present at 2139 cm^−1^.

### 2.2. MIR Spectra of Adsorbed H_2_O

**Figure 4:**
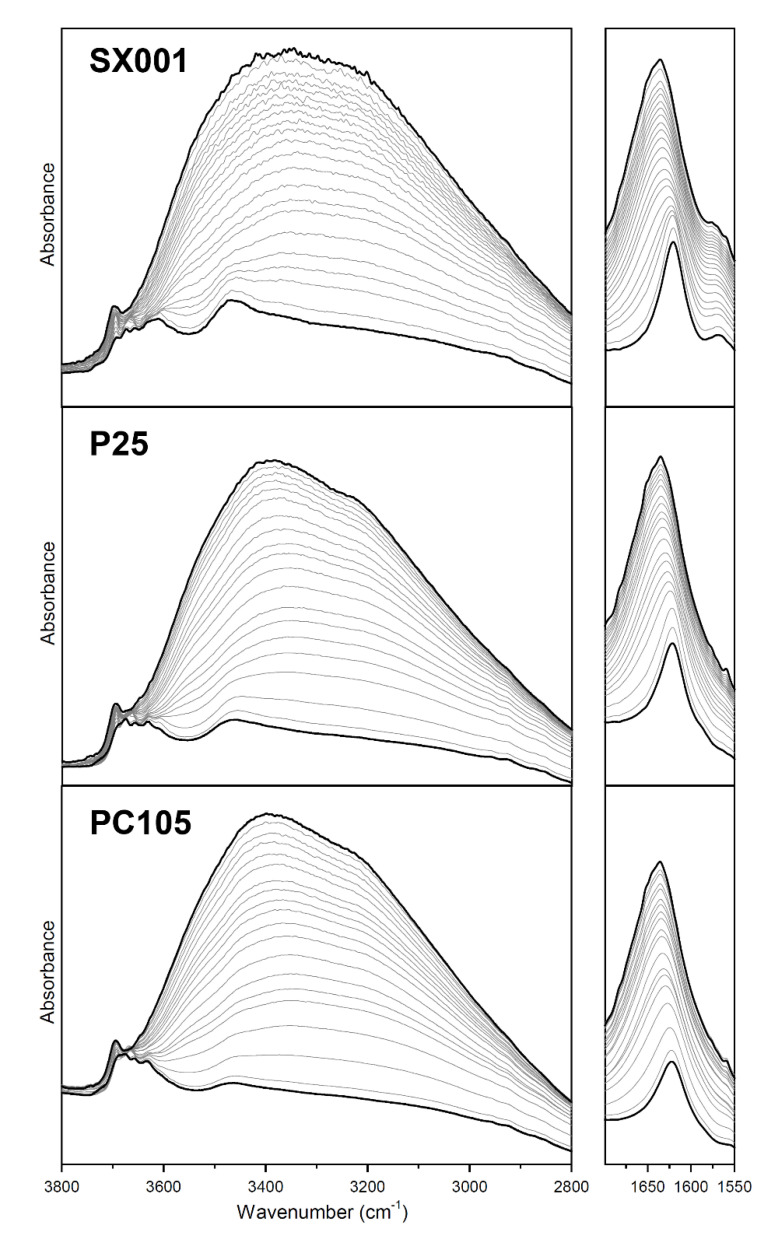
MIR spectra of the different materials, after activation at 873 K, in contact with H_2_O at 15 mbar (curve a) and progressive decreasing of the water coverage till outgassing H_2_O for 15 min at beam temperature (curve s).

A systematic investigation of the adsorption of molecular water on the activated TiO_2_ surfaces has been performed dosing-controlled pressures of water on the different samples. Figure 4 shows the progressive desorption of water starting from H_2_O at 15 mbar to outgassing for 15 min at beam temperature for the three investigated samples. We can note that, at maximum water coverage, the MIR spectra of adsorbed water at 15 mbar on the different samples do not evidence huge differences. Furthermore, they show also similar water desorption isotherms. The broad absorption band in the 3600–2800 cm^−1^ range, visible at the maximum coverage, can be assigned to the ν(OH) of water molecules and hydroxyls interacting via hydrogen bond, while the signal at 3698 cm^−1^ is due to free OH groups of H_2_O molecules pointing out from the water surface multilayer [31,32]. When the coverage is progressively lowered, the intensity of the broad band in the 3600–2800 cm^−1^ range decreases. In parallel, new signals ascribed to free hydroxyl groups start to appear when the water molecules interacting via hydrogen bond are removed. Considering the low frequency region, we can notice that the band due to the water bending mode is centered at 1636 cm^−1^ at maximum coverage and progressively downshifts to 1622 cm^−1^ upon outgassing. This is due to the fading out of the water-water interactions moving from multilayers of adsorbed H_2_O to the monolayer.

**Figure 5:**
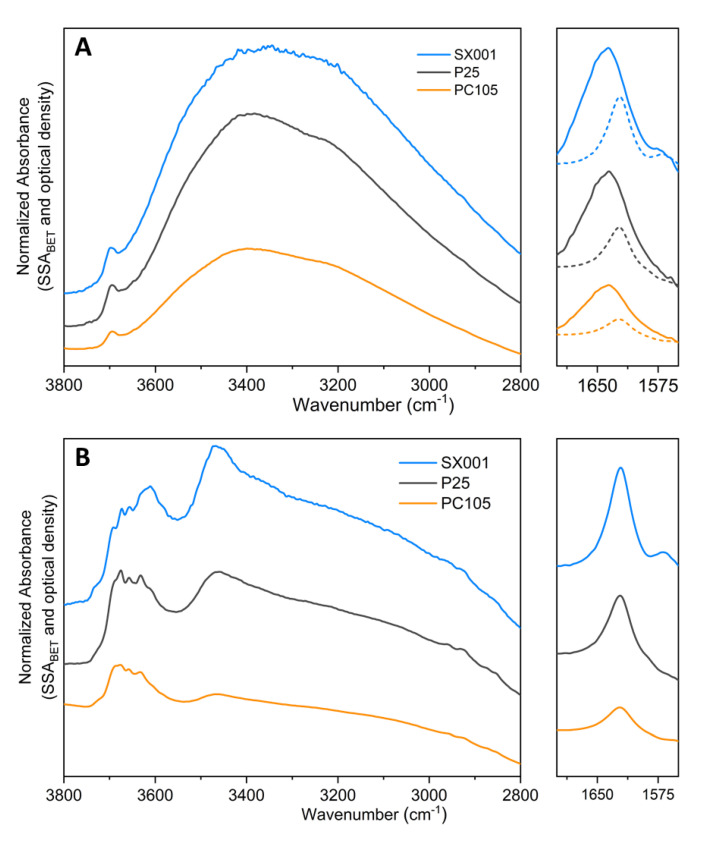
MIR spectra of SX001 (light blue), P25 (dark grey) and PC105 (orange), previously activated at 873 K, in contact with H_2_O at 15 mbar (part **A**) and after outgassing H_2_O for 15 min at beam temperature (part **B**). To facilitate the comparison, the spectra after outgassing for 15 min are also reported in part (**A**), as dashed curves, in the H_2_O bending region. All the spectra have been normalized by dividing the absorbance by the specific surface area (m^2^/g) and the “optical thickness” (g/m^2^) of the pelletized samples.

Considering the MIR spectra normalized for the BET surface area and pellet thickness reported in Figure 5, we can perform a more quantitative comparison on the amount of adsorbed water present on the different samples, which, at maximum coverage follows the trend SX001 > P25 > PC105 (Figure 5A). This trend is confirmed also after outgassing for 15 min (Figure 5B). Nonetheless, in this case, the MIR spectra of the samples show significant differences in the relative intensity of ν(OH) signals centered at 3695, 3675, 3658, 3631, and 3609 cm^−1^ and of the broad band at ca. 3465 cm^−1^. In particular, the bands at 3609 and 3465 cm^−1^ seem to be correlated, as their intensity grows in parallel in the order: SX001 > P25 > PC105, following the same trend of the band related to the water bending mode at 1622 cm^−1^. This observation suggest that these signals could be ascribed to the ν(OH) of H_2_O molecules strongly coordinated with Ti^4+^ sites, likely exposed by {101} surfaces [33]. These are known to adsorb water in molecular form and are the most abundant facets in the NPs according to the CO adsorption results (see Figure 3).

### 2.3. NIR Spectra of Adsorbed H_2_O

**Figure 6:**
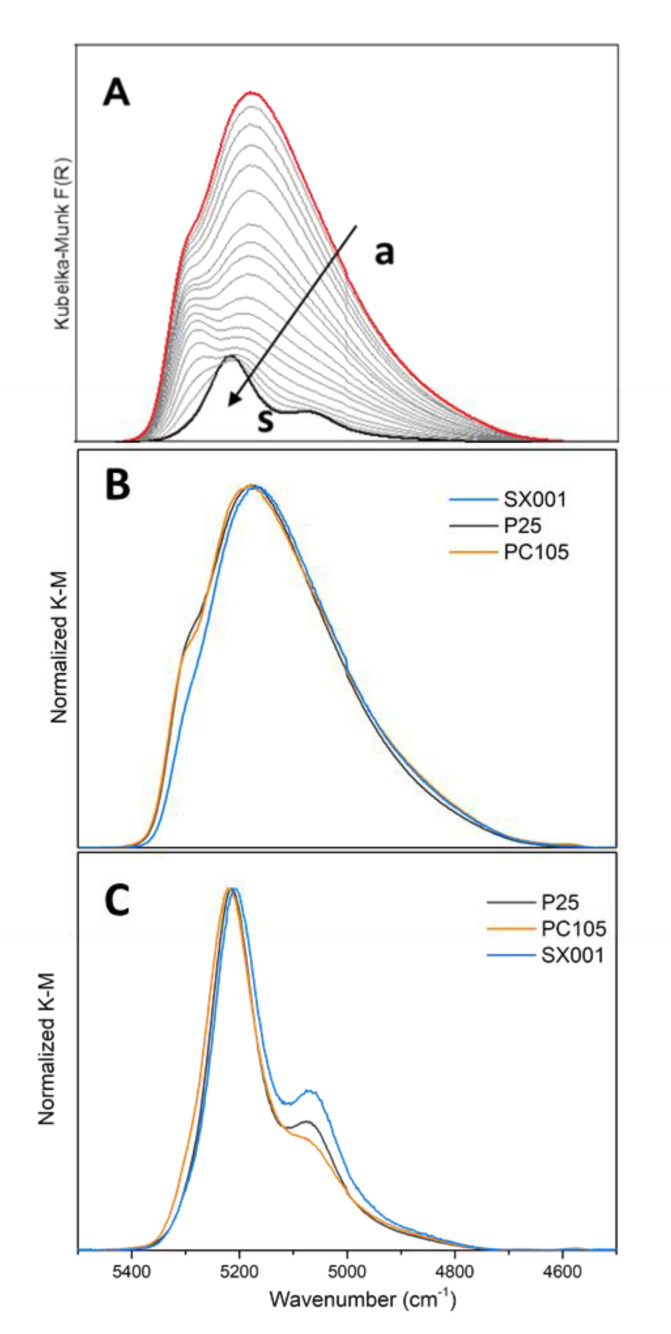
NIR spectra after activation at 873 K of (**A**) P25 in contact with H_2_O at 15 mbar (curve a) and progressive decreasing of the water coverage till outgassing H_2_O for 15 min at room temperature (curve s). (**B**) H_2_O adsorbed at 15 mbar on SX001 (light blue), P25 (dark grey) and PC105 (orange). (**C**) outgassing H_2_O for 15 min at room temperature for on SX001 (light blue), P25 (dark grey) and PC105 (orange). In part (**B**) and (**C**) the spectra have been normalized to the most intense peak.

A detailed analysis of the structure of adsorbed water layers at high coverage by MIR spectroscopy is troublesome, especially in the ν(OH) region where several signals of hydroxyls and hydrogen bonded H_2_O molecules overlap, resulting in a very broad band (Figure 4 and Figure 5), affected also by Fermi resonance contributions [34]. Near-infrared (NIR) spectroscopy is a very useful tool to better address this issue. In fact, in this spectral region, the resolution of each component associated to combination bands of hydrogen-bonded water molecules, is much higher compared to the corresponding fundamental modes observed in the MIR region. Figure 6A shows the NIR spectra of progressive desorption of water starting from H_2_O at 15 mbar (curve a) to outgassing for 15 min at room temperature (curve s) for the P25 sample. The signals in the 5400–4800 cm^−1^ range arise from the combination of H_2_O bending and asymmetric stretching modes (δ + ν_asym_) [34]. This spectral region can be particularly informative on the state of molecularly adsorbed water, because generally the δ + ν_asym_ mode is the most intense with respect to the other combination modes and does not suffer from significant overlap with any components due to surface hydroxyls. Moreover, the contribution from the H_2_O ν_asym_ mode makes this signal sensitive to the interactions experienced by water molecules as H-bonding donors [31].

Thus, considering the spectra at higher coverages in Figure 6, we can assign the different observed signals [31,34]: (i) the contribution at ca. 5300 cm^−1^ is ascribed to adsorbed water molecules acting only as hydrogen bond acceptors, forming the outermost shell of the H_2_O surface multilayer; (ii) the broad band centered at 5180 cm^−1^ contains both the contribution due to H_2_O molecules with one hydroxyl involved in H-bond donation and to H_2_O molecules involved in similar H-bond donation on each hydroxyl, with or without bonding to the oxygen; (iii) the tail extending down to 4700 cm^−1^ arises from water molecules acting as simultaneous donors and acceptors of H-bonds.

While decreasing the water coverage until outgassing for 15 min at room temperature (curve s in Figure 6A), the different components undergo complex modifications due to the fading out of the water-water interactions moving from multilayers of adsorbed H_2_O to the monolayer. In particular, the component at ca. 5300 cm^−1^ and the tail in the 4900–4700 cm^−1^ range disappear, attesting to the removal of the water multilayer. Conversely, the decrease in intensity of the main broad band is accompanied by the formation of two defined components at 5218 and 5068 cm^−1^, tentatively assigned to families of water molecules coordinated to surface cations experiencing weaker and stronger hydrogen bond interactions, respectively.

Figure 6 also reports a comparison of the NIR spectra for the different samples collected when the samples are in contact with water at 15 mbar (part B) and after outgassing H_2_O for 15 min at room temperature (part C), normalized to the most intense peak for sake of comparison. At high water coverage, the main difference is the relative intensity of the component at ca. 5300 cm^−1^, which is related to the sample hydrophobicity. The intensity of this component follows the order SX001 < PC105 ≈ P25, highlighting a higher hydrophilicity of SX001, in agreement with the MIR data.

Considering the normalized NIR spectra upon 15 min of outgassing at room temperature (Figure 6C), we can see that the shape and position of the main band at 5218 cm^−1^ is quite similar for all the TiO_2_ samples, except a small shift to higher frequencies for the PC105. On the contrary, there is a variation of the relative intensity of the component at ca. 5070 cm^−1^, in the order SX001 > P25 > PC105, thus indicating a higher presence of strong hydrogen bond interactions in the SX001 sample. This observation is in agreement with the MIR spectra which show a higher intensity of the bands related to the water bending mode and to the ν(OH) at 3465 cm^−1^ upon outgassing for 15 min at room temperature (see Figure 5).

### 2.4. Determination of Adsorbed H_2_O by Microgravimetry

**Figure 7:**
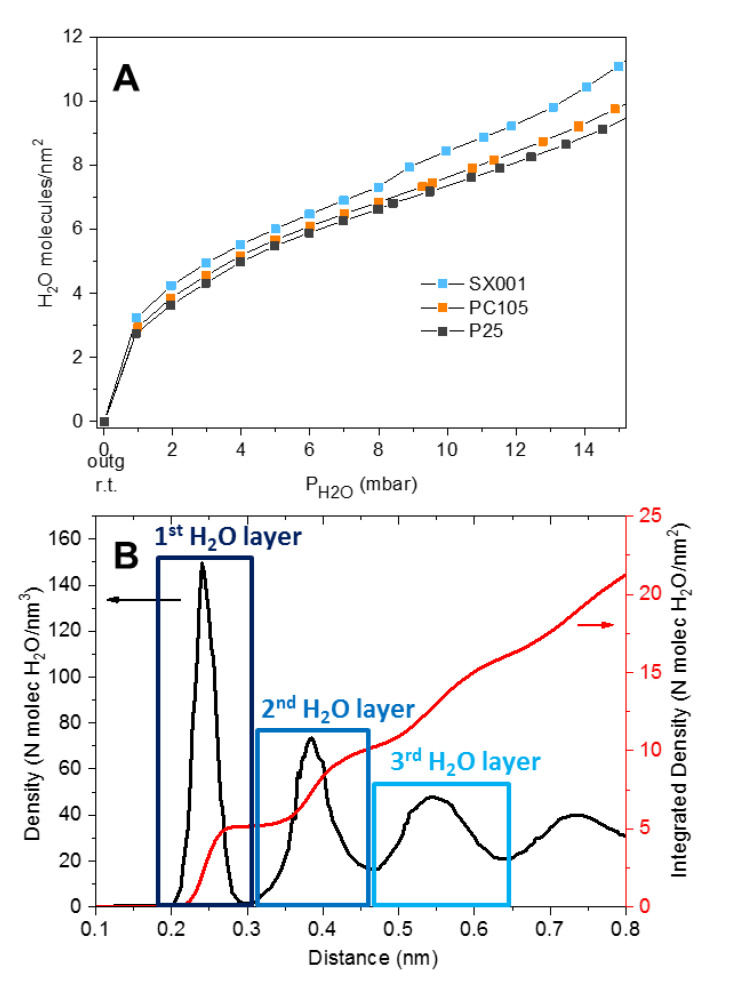
(**A**) Water adsorption isotherms at 298 K obtained from microgravimetric measurements. The samples have been previously outgassed at room temperature (r.t.). (**B**) Water density profile (black curve) and corresponding integration (red curve) in the direction normal to the (101) TiO_2_ anatase surface (the plane formed by the topmost surface titanium atoms is set as 0). The molecular dynamics simulation data (black curve) have been extracted from Figure 3b of ref. [35].

A more quantitative investigation of the amount of H_2_O molecules that can be reversibly adsorbed at room temperature (r.t.) was performed by microgravimetry (Figure 7A). This amount depends on the adsorptive properties of the “first hydroxylation/hydration layer”, constituted by surface OH groups and strongly adsorbed water molecules, which cannot be desorbed by outgassing at r.t., as highlighted also by the MIR (Figure 4 and Figure 5) and NIR (Figure 6) measurements. Figure 7A shows that, in agreement with the spectroscopic data, SX001 is the more hydrophilic sample since it can reversibly adsorb up to ~11 H_2_O molecules/nm^2^ when contacted with H_2_O at 15 mbar. Conversely, P25 and PC105 can adsorb only ~9.5 H_2_O molecules/nm^2^ in the same conditions. If we compare these results with the calculated water density profiles, obtained from molecular dynamics (MD) simulations [35,36], we can estimate the number of water layers formed at the TiO_2_ interface when varying H_2_O equilibrium pressures. For our analysis we will consider the MD profile for water on anatase (101), which is by far the most abundant surface in our samples and it is known to adsorb water in molecular form. We will also assume that the first hydration layer, which from the MD results (Figure 7B) correspond to ~5 H_2_O molecules/nm^2^, is irreversibly adsorbed at room temperature. Considering these assumptions, we can estimate that the second H_2_O layer (corresponding to ~10 H_2_O molecules/nm^2^ in the integrated density of Figure 7B) is completed when the samples are in equilibrium with H_2_O at ~4 mbar. Then, at the maximum investigated water equilibrium pressure (15 mbar), three H_2_O monolayers should be approximately adsorbed at the NPs surface.

## 3. Materials and Methods

### 3.1. Materials and Preliminary Characterization

The following commercial TiO_2_ materials have been selected: (i) TiO_2_ P25, a benchmark in photocatalytic studies, produced by Evonik via flame pyrolysis of TiCl_4_, ~85% anatase and ~15% rutile, specific surface area (obtained applying the Brunauer-Emmett-Teller equation, vide infra): 42 m^2^ g^−1^; (ii) SX001, produced by Solaronix via a sol-gel procedure, employed for dye-sensitized solar cells, pure anatase phase, specific surface area: 43 m^2^ g^−1^; (iii) CristalACTiV™ PC105, produced by Cristal via TiCl_4_ hydrolysis in presence of H_2_SO_4_, pure anatase, specific surface area: 82 m^2^ g^−1^. The specific surface area has been determined after thermal treatment at 873 K, with the same activation protocol performed before spectroscopic measurements (vide infra).

Adsorption experiments of N_2_ at 77 K were carried out by a Micromeritics ASAP 2020 sorption analyzer to measure the specific surface area of the samples. Before the measurements, the samples were outgassed until the attainment of a residual pressure of 1 × 10^−3^ mbar. Data were treated by applying the Brunauer-Emmett-Teller (BET) equation.

High resolution transmission electron microscopy (HR-TEM) images of the materials (powder grains “dry” dispersed on lacey carbon Cu grids) were obtained using a JEOL 3010-UHR microscope operated at 300 kV.

### 3.2. Microgravimetry

Microgravimetric H_2_O adsorption experiments were performed using a Hiden intelligent gravimetric analyzer (IGA). For each measurement, about 100 mg of TiO_2_ powder were transferred into the microbalance equipped with a steel reactor which allows performing adsorption/desorption isotherms at variable temperature. All the treatments (outgassing and water vapor adsorption) were carried out until sample weight equilibrium was reached.

### 3.3. Mid-IR (FTIR) Spectroscopy

An aliquot of each type of TiO_2_ nanoparticles was pressed in self-supporting pellets (“optical density” of ca. 10 mg·cm^−2^) and placed in quartz cells equipped with KBr windows designed to carry out spectroscopic measurements at beam temperature (b.t.; ca. 323 K) or at low temperature (i.e., ~100 K) by cooling with liquid N_2_ [26]. The cells were connected to a conventional vacuum line (residual pressure: 1 × 10^−5^ mbar) allowing all thermal treatments and adsorption-desorption experiments to be carried out in situ. A Bruker IFS 28 spectrometer (resolution: 2 cm^−1^; detector: MCT) was employed for the spectra collection, averaging 128 scans. Before dosing the desired molecule (CO or H_2_O), the TiO_2_ samples were activated with the following procedure: (i) outgassing at 873 K for 120 min; (ii) contacting with 20 mbar of O_2_ at the same temperature; (iii) cooling to 373 K in O_2_; and (iv) cooling from 373 K to r.t. under outgassing. 

### 3.4. Near IR Spectroscopy

Diffuse reflectance (DR) NIR spectra were recorded with a Cary 5000 Varian spectrophotometer equipped with an integrating sphere with an inner coating of Spectralon^®^. This material was used also as reference. For the collection of spectra, thick self-supported pellets of TiO_2_ nanoparticles were placed in a cell with an optical quartz window designed to carry out the measurements in controlled atmosphere, by connection to a conventional vacuum line. Before dosing H_2_O, the TiO_2_ samples were activated with the same procedure employed for Mid-IR spectroscopy.

## 4. Conclusions

In this contribution, we performed a systematic investigation of particle morphology, surface properties, and hydration state for three different commercial TiO_2_ samples. The HR-TEM images highlight that all samples show some truncated bipyramidal nanoparticles, exposing {101} facets, but their size and shape distribution is heterogeneous and their borders are quite irregular. Therefore, it is difficult to draw definitive conclusions from the electron microscopy data. A more detailed investigation by MIR spectroscopy of adsorbed CO highlights a similar distribution of surface sites for all samples, confirming that the main exposed facet is anatase {101} for all the investigated NPs, as expected from the Wulff equilibrium shape for anatase TiO_2_. This observation underlines that vibrational spectroscopy of adsorbed probe molecules can provide useful information about the different exposed surfaces, even for heterogeneous samples, where the extraction of statistically significant data from electron micrographs is troublesome.

The combination of MIR and NIR spectroscopies allowed us to study the evolution of the surface hydration state at different water equilibrium pressures, monitoring the progressive fading out of the water-water interactions moving from multilayers of adsorbed H_2_O to the monolayer. Both at the maximum water coverage and after outgassing at room temperature, the amount of molecular water adsorbed on the samples followed the same trend, SX001 > P25 > PC105. This trend could be correlated with the higher regularity of {101} facets (mainly responsible for the adsorption of water in molecular form) in the SX001 sample, as highlighted by IR spectroscopy of the adsorbed CO. Conversely, the PC105 sample showed a higher content of minority facets and Ti centers with high coordinative unsaturation.

Finally, by combining microgravimetric and molecular dynamics results, we estimated the adsorbed water content at increasing H_2_O equilibrium pressures. In particular, we determined that the second layer of adsorbed H_2_O is completed when the NPs are in equilibrium with H_2_O at 4 mbar and that, at 15 mbar, about three complete H_2_O monolayers are formed at the oxide surface.
